# THE CONTRIBUTION OF SELF-REPORTED COGNITIVE HEALTH FOR INFORMING DAILY WELL-BEING AND FUNCTION ACROSS CONTEXTS

**DOI:** 10.1093/geroni/igae098.1974

**Published:** 2024-12-31

**Authors:** Robert Stawski, Kelly Cichy, Stacey Scott

**Affiliations:** Chair; Co-Chair; Discussant

## Abstract

Intensive assessment of experiences, health, and well-being in daily life have shown considerable value for understanding how our experiences shape proximal as well as distal health outcomes throughout midlife and older age. To date, stressors have been the most-oft studied experiences, while negative affect and physical symptoms have been focal indicators of daily well-being. Emerging research is emphasizing the import of experiences across different contexts, including social support provision and the work-family intersection, for shaping health and well-being. Further, self-reports of cognitive health (e.g., memory failures, cognitive complaints, cognitive interference, perseverative cognition) are emerging as an important dimension of daily well-being. Such reports are indicative of stress responses, relevant anxiety and depression symptomology, and dementia risk. Thus, self-reports of cognitive health represent a potential pathway for understanding links among daily experiences and health. The purpose of this symposium is to bring together these diverse operationalizations of cognitive health in daily life to illustrate its value and utility across different daily life experiences and contexts. Cichy and colleagues examine associations among daily social support provision and cognitive interference between persons and within persons across days. Chandler and colleagues examine associations between work-family conflict, cognitive interference, and memory failures among midlife adults in the Work-Family Health Study. Mogle explores age differences in individuals’ attributions for why they could not complete tasks in daily life. Stawski and colleagues examine associations among daily stressors, perseverative cognition, and mortality. Stacey Scott will facilitate discussion of the papers and offer insights for future research.

